# Supplementary material for: Learning to Express Reward Prediction Error-like Dopaminergic Activity Requires Plastic Representations of Time
Source: Res Sq. 2023 Sep 19:rs.3.rs-3289985. Preprint. [Version 1] doi: 10.21203/rs.3.rs-3289985/v1 (PMC10543312; doi:10.21203/rs.3.rs-3289985/v1)
Supplement: Supplement 1 [file NIHPPRS3289985V1-supplement-1.pdf]

Supplemental Figures

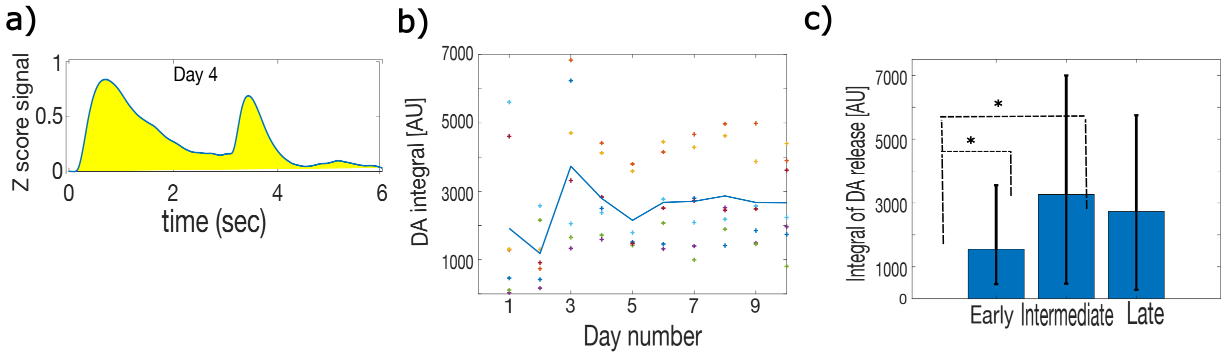

Supplemental Figure 1 – Dynamics of the DA integral.

a) Time course of Z scored signal per one animal and one day from the Amo et al data (2022)[23] (blue curve). The yellow shaded area illustrates the definition of the integrated signal. **b)** Re-analyzed data from Amo et al (2022)[23]. Here we plot the time evolution of the integral of DA response over training days. The different color symbols are the average DA integral (averaged over trials) per each training day, per each animal. Different animals are color coded. The blue line is the mean over animals. **c)** Average integrals for early (days 1-2), intermediate (days 3-4) and late (days 8-10) of training. Early is significantly lower than intermediate (Wilcoxon rank sum test p=0.004) and late (p=0.006), but intermediate is not significantly higher than late.

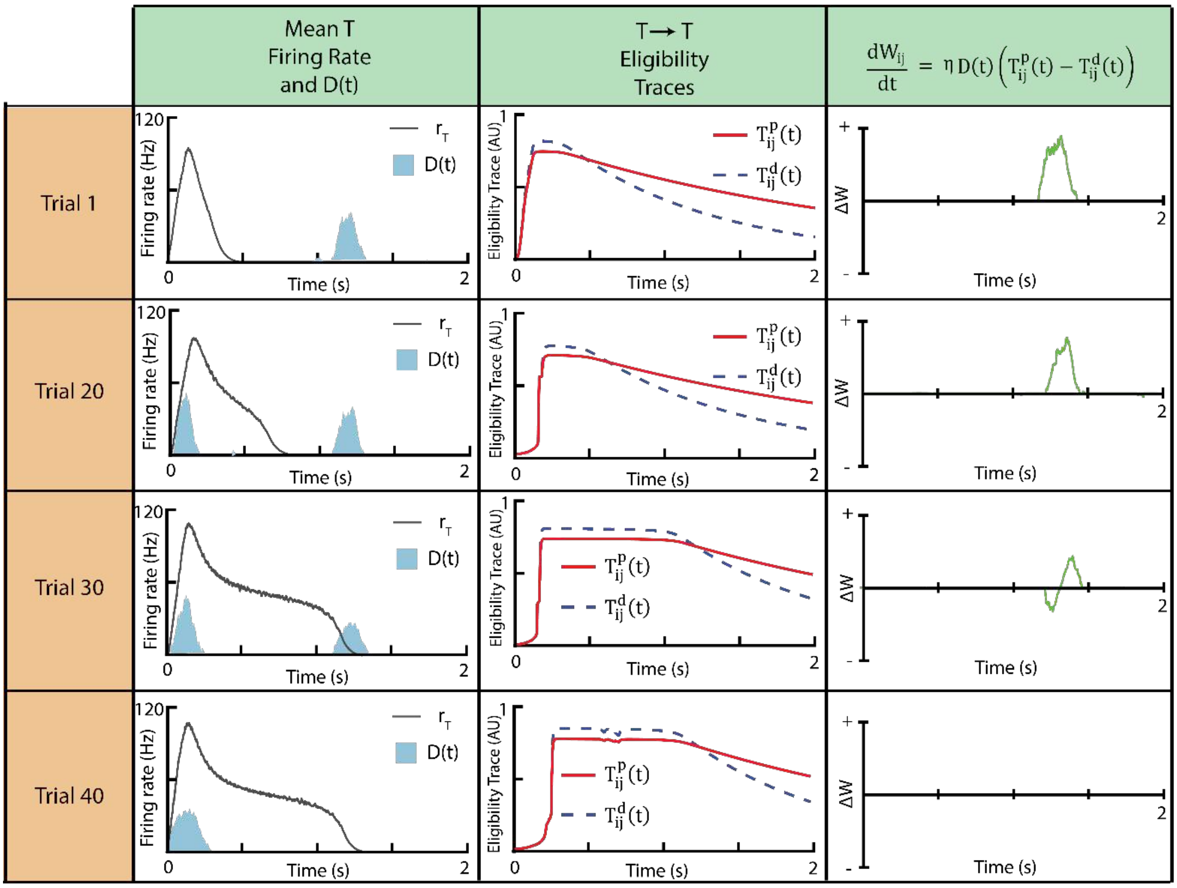

Supplemental Figure 2 – Two-Trace Learning Flexibly Encodes Cue-Reward Delay in Recurrent Connections of Timer Neurons Demonstration of the dynamics of two-trace learning for recurrent connections. Rows consist of different trials. Left column, mean firing rate of Timer neurons (black) and dopamine reinforcement D(t) (light blue) for a given trial. Middle column, LTP (red) and LTD (blue) associated eligibility traces triggered by the Hebbian overlap $\frac{r_i \cdot r_j}{1+\alpha D(t)}$. Right column, $\frac{dW}{dt}$, calculated at a given time as the difference between the two traces $(T_{ij}^{p} - T_{ij}^{d})$ multiplied by the dopamine reinforcement, D(t). For all trials, the increase in recurrent weights is mediated by dopamine release at t_{US}. Since dopamine acts to suppress trace generation in PFC, the CS-evoked dopamine response (trial 20) has little effect on the recurrent learning. The weights increase until ΔW is zero (trial 30) and remain at their fixed point after US-evoked dopamine has been suppressed (trial 40).

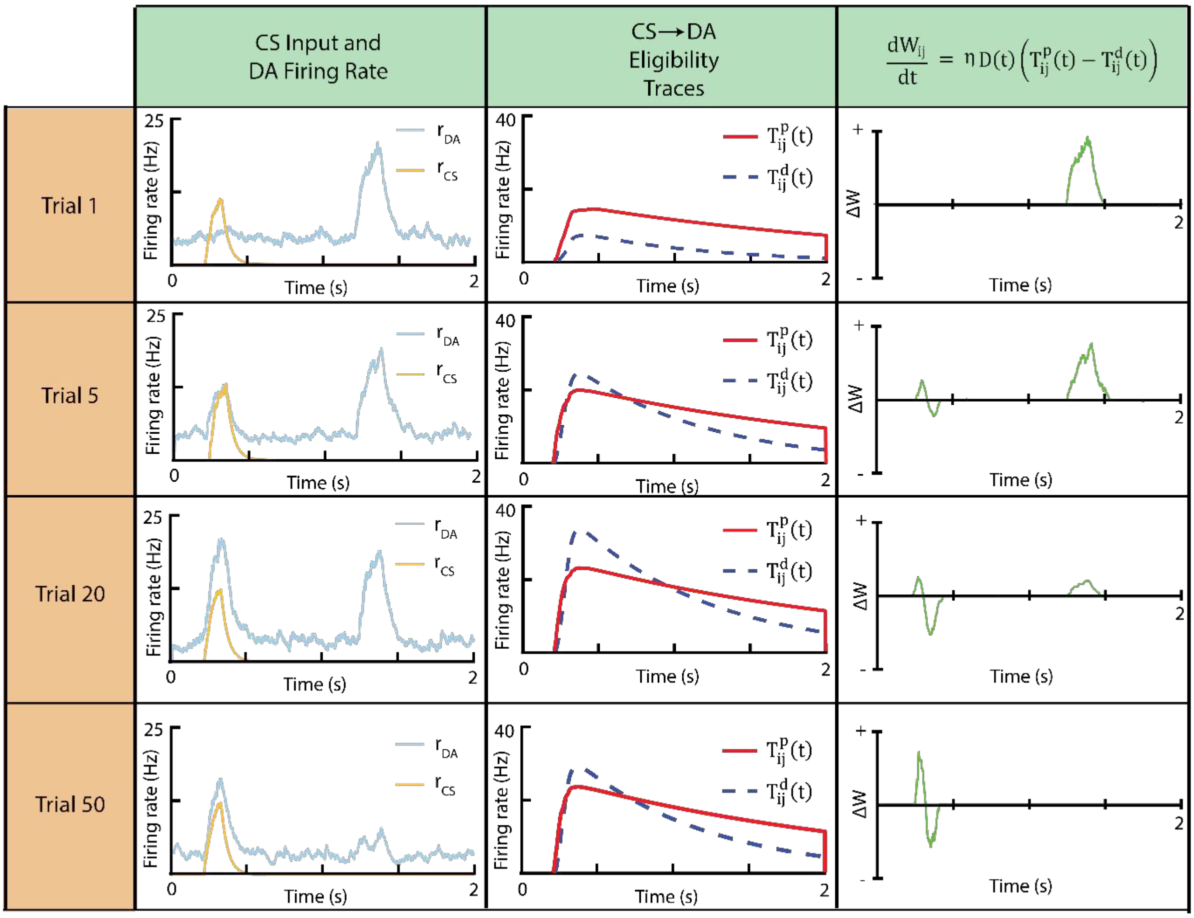

Supplemental Figure 3 – Two-Trace Learning Encodes Reward Predictive Cues via Feed-Forward Connections to DA Neurons

Demonstration of the dynamics of two-trace learning for feed-forward connections. Rows consist of different trials. Left column, input from external conditioned stimulus (orange) and mean firing rate of dopamine neurons (light blue) for a given trial. Middle column, LTP (red) and LTD (blue) associated eligibility traces triggered by the Hebbian overlap $r_{CS} * r_{DA}$. Right column, $\frac{dW}{dt}$, calculated at a given time as the difference between the two traces ($T_{ij}^{p} - T_{ij}^{d}$) multiplied by the dopamine reinforcement, D(t). Initially (trial 1), increase in feed forward weights is mediated by dopamine release at t_{US}. However, as a CS-evoked dopamine response begins to develop (trial 5), the weights are bounded at a fixed point, constrained by the relative positive (from the US) and negative (from the CS) contributions (trial 20). After the expected reward at t_{US} has been depressed, the DA neuron firing at t_{CS} decreases slightly until $\frac{dW}{dt}$ reaches a fixed point maintained by the CS dopamine alone.

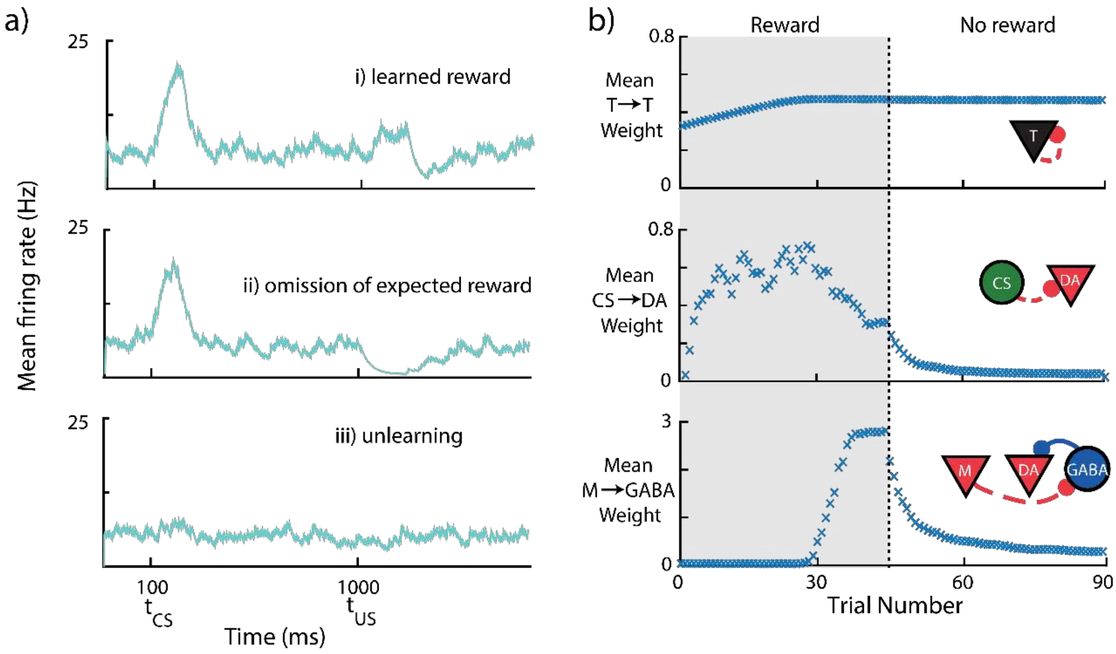

Supplemental Figure 4 – Consistent Unpairing Leads to Negative RPEs and Unlearning

a) Mean firing rate over all VTA DA neurons for a given trial after learning reward (i), upon omission of the learned reward (ii), and finally, following unlearning (iii). The omission of the expected reward produces a characteristic dopamine "dip", which in turn acts as a negative RPE and facilitates unlearning of the cue-reward association. **b)** Mean Timer→Timer (top), CS→DA (middle), and M→GABA (bottom) synaptic weights over the course of pairing and unpairing. The cue is presented for all trials, while the reward is only presented for the first 45 trials (shaded grey). For trials ~30-45, M→GABA weights increase, suppressing the amount of DA firing at the time of the reward. In response, the fixed point of CS→DA weights (which is determined, in part, by the dynamics of the dopamine reinforcement D(t)) decreases before stabilizing in trials ~40-45. Following omission, both CS→DA and M→GABA weights decrease to zero, but T→T weights are maintained, since the fixed point for these weights (see **Supplemental Figure 2**) is agnostic to changes in to D(t).

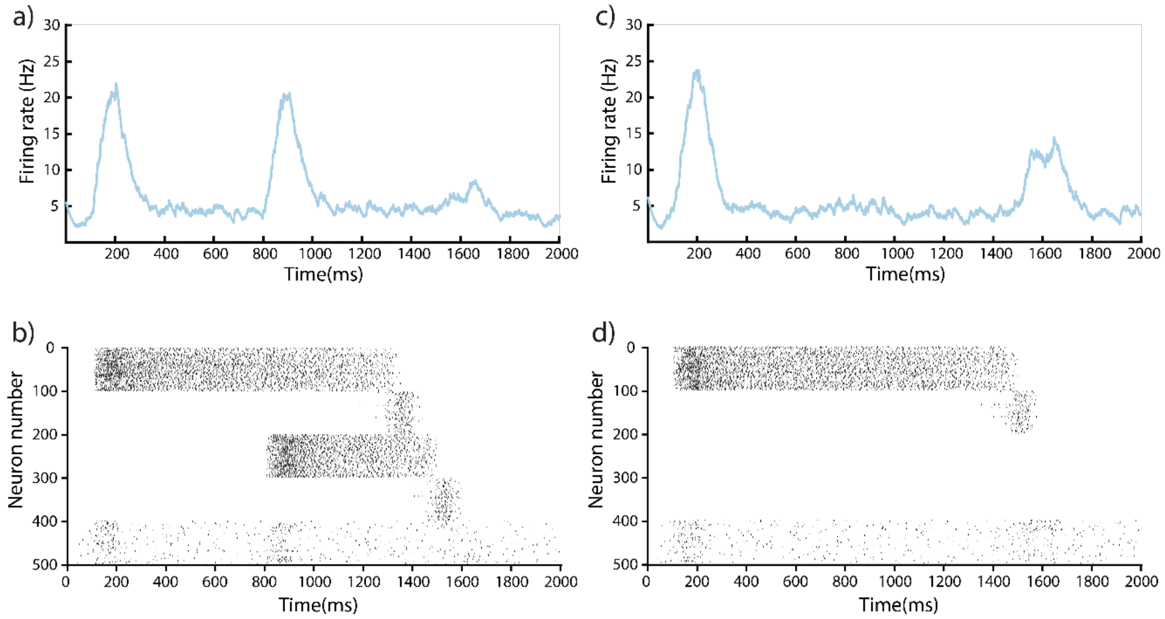

Supplemental Figure 5 – Both Cues Contribute to Reward Prediction Following Initial Training

a) Mean firing rate of all VTA DA neurons for presentation of CS1+CS2+US following sequential conditioning. **b)** Spike raster of Timer neurons (1-100, 201-300), Messenger neurons (101-200, 301-400), and VTA DA neurons (401-500) for the same trial. **c)** Mean firing rate of all VTA DA neurons for presentation of CS1+US, omitting CS2 following simultaneous conditioning. Notably, CS1 only partially predicts (and in turn partially inhibits) the dopamine response at t_{US}. d**)** Spike raster of Timer neurons (1-100, 201-300), Messenger neurons (101-200, 301-400), and VTA DA neurons (401-500) for the same trial.

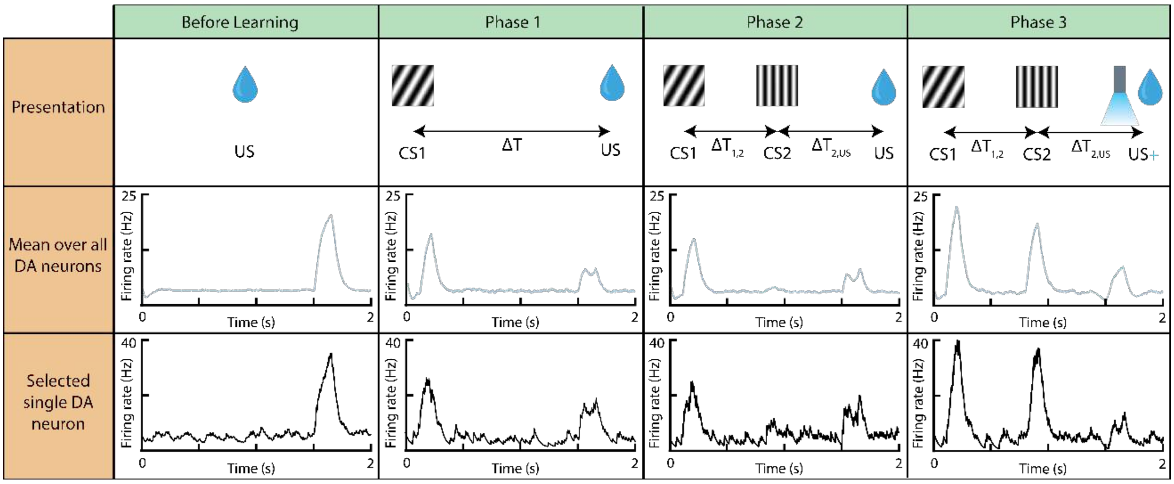

Supplemental Figure 6 – Expected Rewards Block Learning of New Cue-Reward Associations Unless Reward Magnitude is Increased

Each column marks a different phase of conditioning in a blocking/unblocking paradigm. Results shown are averaged over 25 trials. Top row, visual representation of protocol in given column. Middle row, mean over all DA neurons and trials for given presentation protocol. Bottom row, a selected single unit response averaged over trials of the given presentation protocol. Before learning, unpredicted rewards trigger dopamine release at t_{US}. In phase 1, CS1→US is learned, and DA neurons develop a response to CS1 and have suppressed their response to the US. In phase 2, CS1→CS2→US is presented, but a dopamine response fails to develop to CS2, as the US is already fully predicted by CS1 (and therefore CS2 is "blocked"). Phase 3 results in "unblocking" via increasing dopamine release at t_{US} – doing so recovers the CS2-evoked response and results in both stimuli becoming reward-predictive.
